# Supplementary material for: Pseudomonas putida mediates bacterial killing, biofilm invasion and biocontrol with a type IVB secretion system
Source: Nat Microbiol. 2022 Sep 19;7(10):1547–57. doi: 10.1038/s41564-022-01209-6 (PMC9519443; doi:10.1038/s41564-022-01209-6)
Supplement: Supplementary file 1 — Supplementary Figs. 1–6, Tables 1–6 and references. [file 41564_2022_1209_MOESM1_ESM.pdf]

nature portfolio

https://doi.org/10.1038/s41564-022-01209-6

Supplementary information

Pseudomonas putida mediates bacterial killing, biofilm invasion and biocontrol with a type IVB secretion system

In the format provided by the authors and unedited

***Pseudomonas putida* mediates bacterial killing, biofilm invasion and biocontrol with a type IVB secretion system**

Purtschert-Montenegro *et al.*

Supplementary information

Supplementary Figures S1 to S6

Source Data File – Supplementary Figure 1

Source Data File – Supplementary Figure 6

Supplementary Tables S1 to S6

Supplementary references

Source Data Files

Source Data Files - Figures 1 to 6

Source Data File - Extended Data Figure 1

Source Data File - Extended Data Figure 2

Source Data File - Extended Data Figure 5

Source Data File - Extended Data Figure 6

Source Data File - Extended Data Figure 8

Source Data File - Extended Data Figure 9

Supplementary Information

Supplementary Figures

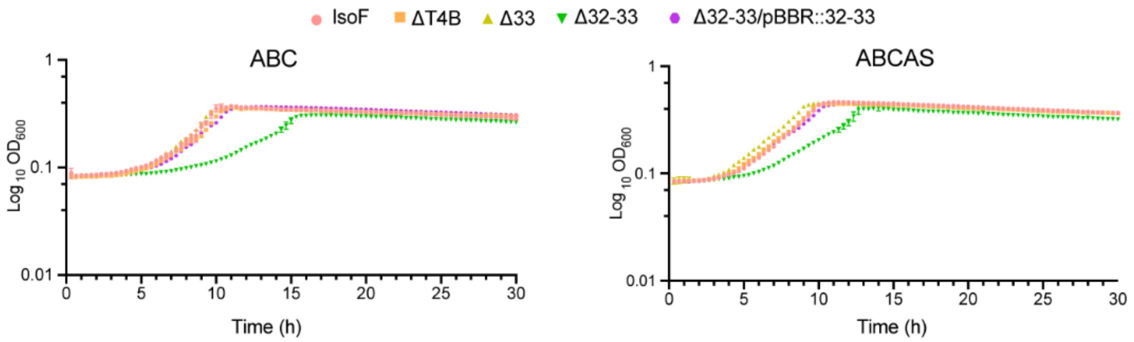

Figure S1. Growth of IsoF and various deletion mutants in ABC minimal medium in the absence or presence of casamino acids (ABCAS). Data are mean ± s.d. of two biological replicates (n=2).

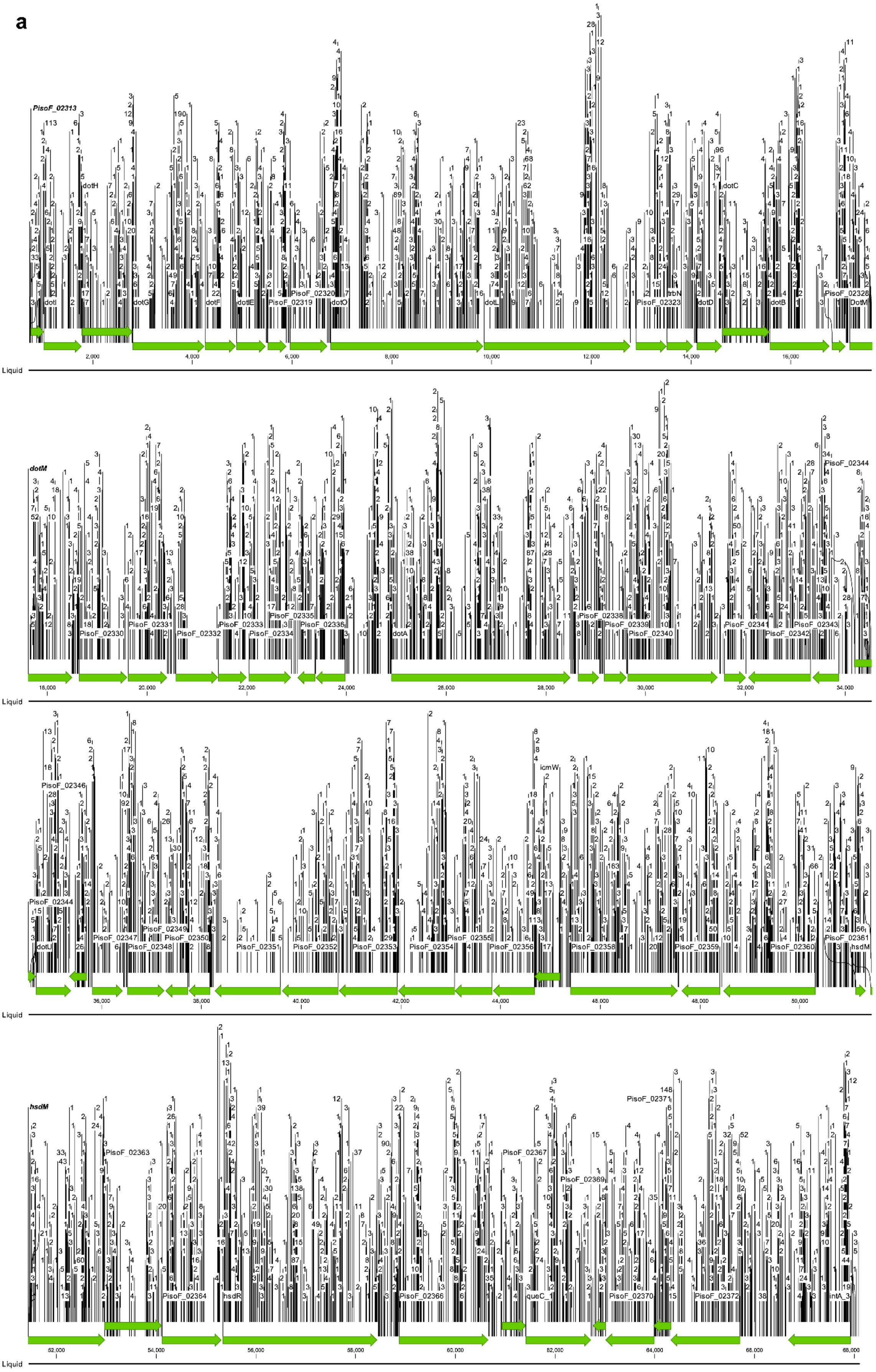

Figure S2. Transposon insertions mapped on the genomic island of IsoF. Growth in liquid medium.

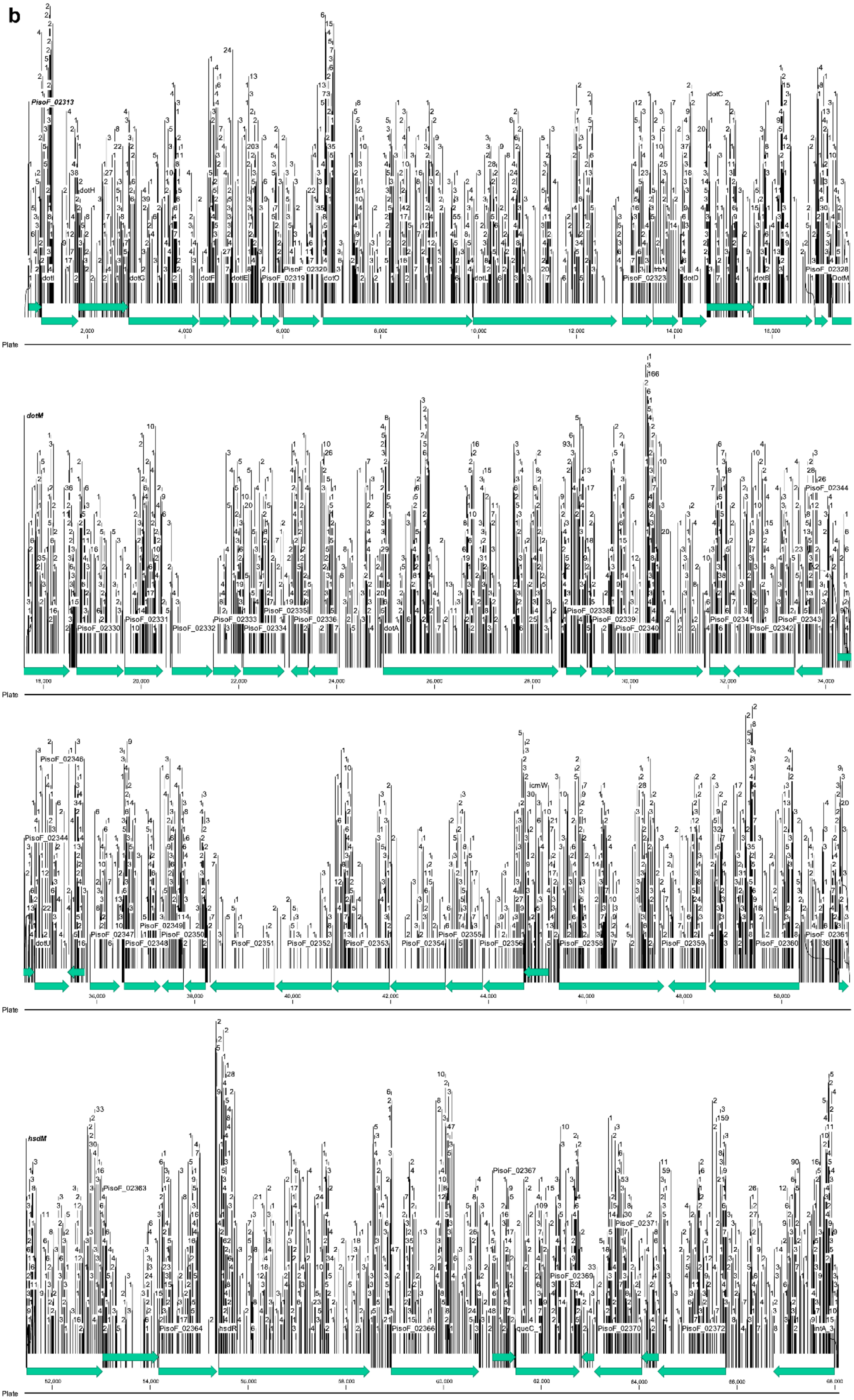

Figure S3. Transposon insertions mapped on the genomic island of IsoF. Growth on an agar surface.

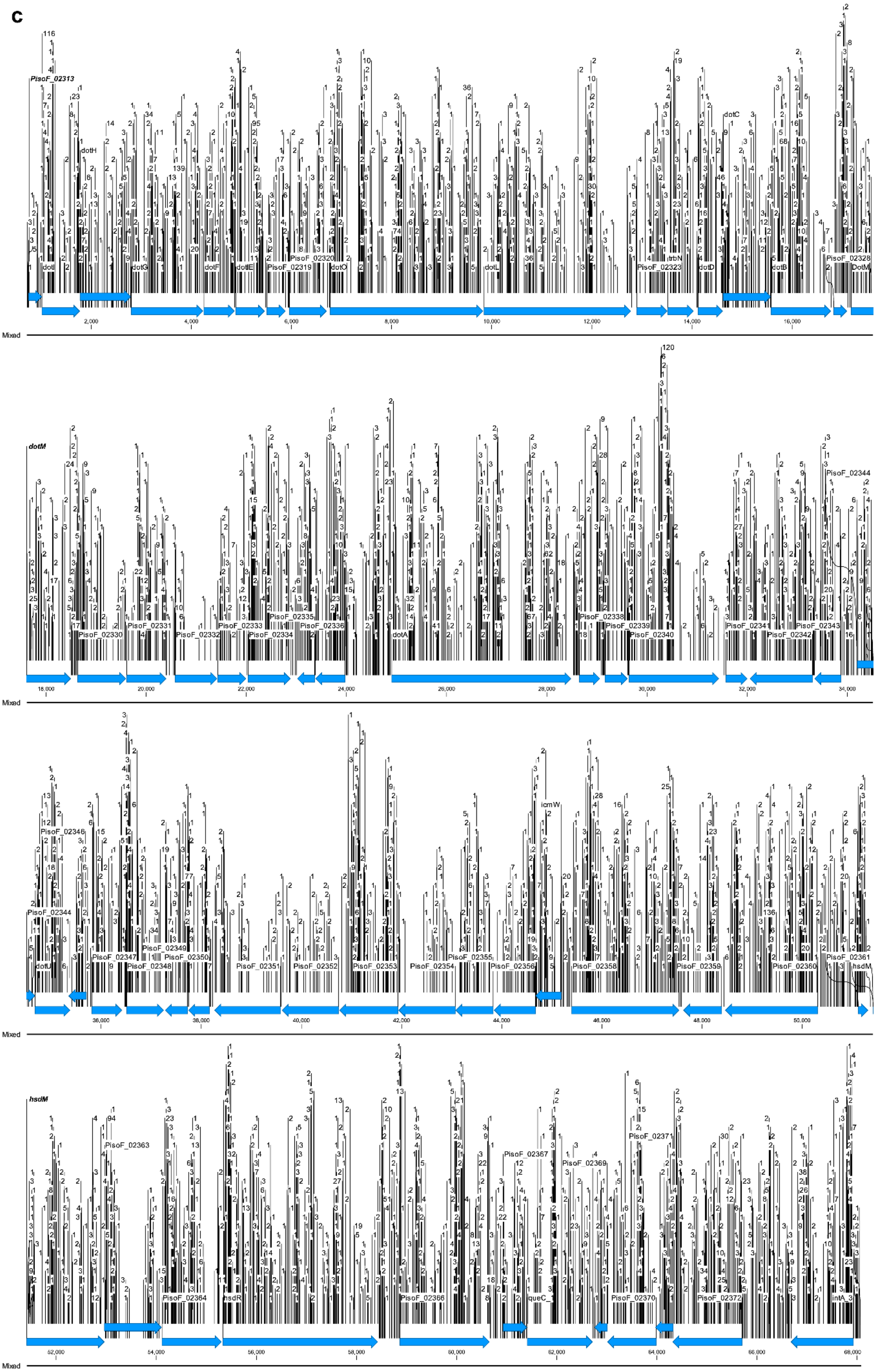

Figure S4. Transposon insertions mapped on the genomic island of IsoF. Growth on an agar surface in the presence of *P. aureofaciens*.

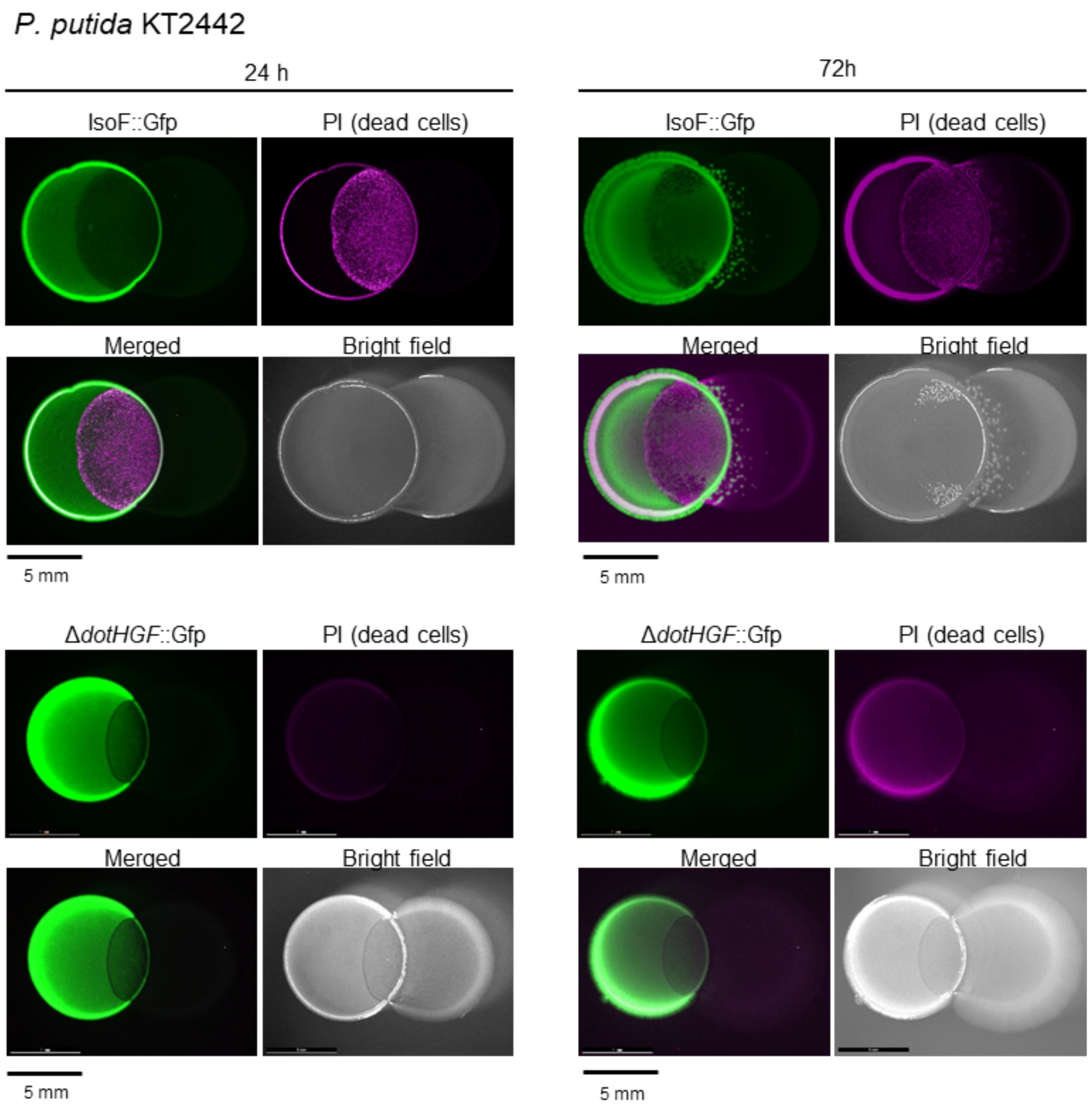

Figure S5. Representative pictures of contact-dependent killing and macrocolony development of KT2442 when co-inoculated with IsoF::Gfp and Δ*dotHGF*::Gfp. The medium was supplemented with propidium iodide to stain dead cells (magenta). Images were taken at 24 and 72 hours after inoculation. Scale bar, 5 mm.

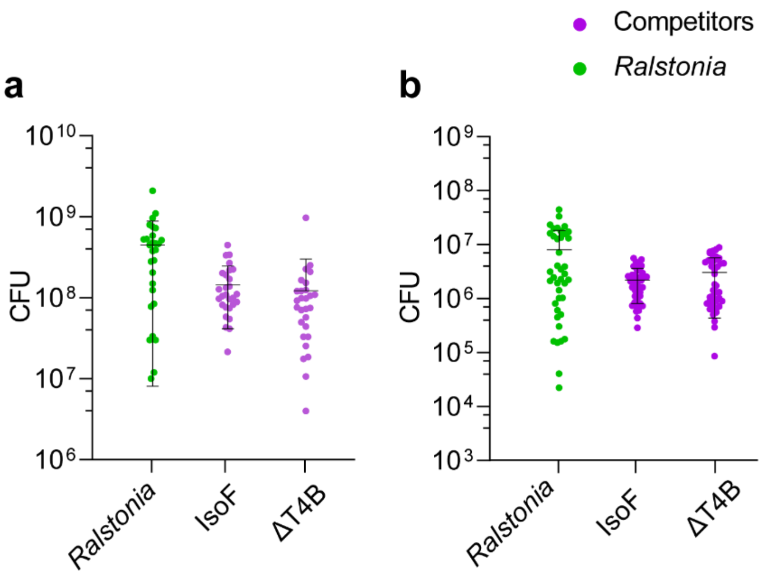

Figure S6. Recovered CFUs from the roots of tomato plants treated with monocultures of *Ralstonia*::Gfp, IsoF::mCherry and ΔT4B::mCherry. **a**, CFUs from plant grown on MS plates. **b**, CFUs from plant experiment grown in non-sterile soil. Error bars show mean ± s.d. (n=3).

Supplementary Tables

Table S1. List of strains used in this study.

Strain or plasmid	Characteristics	Source or reference
**Strains**		
E. coli		
MC1061	hsdR araD139 Δ(ara-leu)7697 ΔlacX74 galU galK rpsL (Sm^R)	Casabadan, et al.,1980[1]
CC118λpir	Δ*(ara, leu)7697 araD*139 Δ*lacX74 galE galK phoA20 thi-1 rpsE rpoB(*Rf^R*) argE(Am) recA1 λpir^R*	Herrero et al., 1990[2]
S17-1λpir	*thi proA hsdR recA* RP4-2-*tet*::Mu-1 *kan*::Tn*7* integrant (Tp^R Sm^R) λpir	Simon, 1983[3]
Dh5α	F- Φ80d*lacZ*Δ*M15* Δ*(lacZYA-argF)*U169 *recA1 endA1 hsdR17(rK$^-$ mK$^+$) supE44 thi-1 relA1 gyrA96*	Hanahan, 1983[4]
SY327λpir	wild type; *araD*, Δ(*lac pro*) *argE*(Am) *recA56* Rif^R *nalA λpir*	Miller et al., 1988[5]
GM2163	*dam*-13::Tn9 (Camr) *dcm-6 hsdR2* (*rK$^-$ mK$^+$)leuB6 hisG4 thi-1 araC14 lacY1 galK2 galT22 xylA5 mtl-1 rpsL136* (Strr) *fhuA31 tsx-78 glnV44 mcrA mcrB1*	Fermentas
P. putida*		
IsoF	wild type; isolated from tomato roots; AHL$^+$	Steidle et al 2001[6]
Δ*dotHGF*	IsoF deletion mutant of *dotH, dotG, dotF*	This study
Δ*dotHGF* /pBBR::32-33	Complementation of *Piso_02332* and *Piso_02333* using pBBR1MCS-2 derivative	This study
Δ23	IsoF deletion mutant of *Piso_02323*	This study
Δ*dotD*	IsoF deletion mutant of *dotD*	This study
Δ23-*trbN*-*dotD*	IsoF deletion mutant of *Piso_02323, trbN, dotD*	This study
Δ32-33	IsoF deletion mutant of *Piso_02332, Piso_02333*	This study
Δ32-33 /pBBR::32-33	Complementation of *Piso_02332* and *Piso_02333* using pBBR1MCS-2 derivative	This study
Δ32-33/pBBR::32	Complementation of Piso_*02332* using pBBR1MCS-2 derivative	This study
Δ33	IsoF deletion mutant of *Piso_02333*	This study
Δ33/pBBR::33	Complementation of *Piso_02333* using pBBR1MCS-2 derivative	This study
ΔT4B	IsoF deletion mutant of the *kib* cluster, from *Piso_02313* to *Piso_02360*	This study

Strains	Characteristics	Source or reference
∆T4B/pBBR::32	Complementation of *Piso_02332* using pBBR1MCS-2 derivative	This study
∆T4B/pBBR::32-33	Complementation of *Piso_02332* and *Piso_02333* using pBBR1MCS-2 derivative	This study
KT2442*	Wild type; *hsdR* Rif^R	Laboratory collection
W2*	wild type	Laboratory collection
OUS82*	wild type	Laboratory collection
A9Rx29*	wild type	Laboratory collection
P. aureofaciens*	ATCC13985; wild type	Conway et al.,1956[7]
P. entomophila*	wild type	Laboratory collection
P. chlororaphis*	wild type	Laboratory collection
P. fluorescens*	WCS417r; wild type	Laboratory collection
P. syringae*	DC3000; wild type	Laboratory collection
Pectobacterium carotovorum*	wild type	Laboratory collection
Ralstonia solanacearum*	DSM9544; wild type	Laboratory collection
P. aeruginosa		
JB646	Wild type, Gfp tagged	Laboratory collection
PUPA3	Carrying pBBR-5-Gfp	Laboratory collection
PA14*	wild type	Rakhimova et al., 2008[8]
PAO1*	wild type	Iglewski et al., 1999[9]

Strains	Characteristics	Source or reference
Burkholderia cenocepacia	H111 tagged with Gfp	Laboratory collection
Burkholderia vietnamiensis	LMG10929 tagged with Gfp	Laboratory collection
Burkholderia cepacia	M64; carrying pBBR-5-Gfp	Laboratory collection
Burkholderia multivorans	R-654 tagged with Gfp	Laboratory collection
Serratia liquefaciens	MG1 tagged with Gfp	Laboratory collection
* These strains and its variants were labeled either with Gfp, mCherry or Cfp using the pUC18T-mini-Tn*7*T-Gm when indicated.		This study
**Plasmids**		
pUC18T-mini-Tn*7*T-Gm-*egfp-a*	carrying mini Tn7(Gm) PA1/04/03-egfp-a for Gfp tagging	Choi and Schweizer, 2006[10]
pUC18T-mini-Tn*7*T-Gm-*mCherry*	carrying mini Tn7(Gm) PA1/04/03-mCherry for mCherry tagging	Choi and Schweizer, 2006
pUC18T-mini-Tn*7*T-Gm-*ecfp*	carrying mini Tn7(Gm) PA1/04/03-ecfp for Cfp tagging	Choi and Schweizer, 2006
pUX-BF13	tnsA-E; helper plasmid providing the Tn7 transposition functions	Koch et al., 2001[11]
pUTminiTn5	delivery vector for mini-Tn*5* Km2; Km^R	De Lorenzo et al, 1990[2]
pGPI-SceI	Suicide plasmid with oriR6K, mob^+, I-SceI restriction site; Tp^R	Elisabeth Steiner, Laboratory collection
pGPI-SceI::TetAR	Suicide plasmid with oriR6K, mob^+, I-SceI restriction site; Tp^R Tc^R	This study
pGPI::Tet-H-F	pGPI-SceI-Tet plasmid with fused regions flanking the genes *dotH, dotG, dotF*	This study
pGPI::Tet-23	pGPI-SceI-Tet plasmid with fused regions flanking the gene *Piso_02323*	This study
pGPI::Tet-DotD	pGPI-SceI-Tet plasmid with fused regions flanking the gene *dotD*	This study

Plasmids	Characteristics	Source or reference
pGPI::Tet-23-DotD	pGPI-SceI-Tet plasmid with fused regions flanking the genes *Piso_02323* and *dotD*	This study
pGPI::Tet-32-33	pGPI-SceI-Tet plasmid with fused regions flanking the genes *Piso_02332* and *Piso_02333*	This study
pGPI::Tet-33	pGPI-SceI-Tet plasmid with fused regions flanking the gene *Piso_02333*	This study
pGPI::TetAR-T4B	pGPI-SceI-Tet plasmid with fused regions flanking the genes *Piso_02313* to *intA_3*	This study
pDAIGm-SceI	pDA17 plasmid carrying the *I-SceI* nuclease gene; Gm^R	Elisabeth Steiner, Flannagan et al., 2008[12]
pRK2013	Helper plasmid; RK2 derivative, *mob*$^+$ *tra*$^+$ *ori* ColE1; Km^R	Figurski *et al.*, 1979[13]
pBBR1MCS-2	Broad-host-range plasmid, *lac*Zα; Km^R	Kovach et al 1995[14]
pBBR::32	pBBR1MCS-2 carrying *Piso_02332*, complementation plasmid	This study
pBBR::33	pBBR1MCS-2 carrying *Piso_02333*, complementation plasmid	This study
pBBR::32-33	pBBR1MCS-2 carrying *Piso_02332* and *Piso_02333*, complementation plasmid	This study
pLG99	Transposon T23 (IS*lacZ*-P*rhaB* out-/FRT); Tp^R	Gallagher et al., 2013[15]
pLG99::Km	Transposon T23 (IS*lacZ*-P*rhaB* out-/FRT); Km^R, Tp^R	This study

Table S2. List of primers used in this study.

Primer name	Sequence	Restriction site/Description
**Primers used for construction of markerless deletion of *dotH, dotG, dotF***		
DotIHindIII_F	GCGCaagcttTGCGTATTCGGCTTGACC	HindIII
DotIXhoI_R	GCGCctcgagGGCGACAGCTGAACAGAA	XhoI
DotIEXhoI_F	GCGCctcgagATAGGGAAATACACATGGC	XhoI
dotIEcoRI_R	GCGCgaattcGGCTTGCGTTTGATTATTG	EcoRI
DotI_out_F	TCGACCTCAAGCGCCGTAA	Check primer
DotIE_out_R	CCGCCAATAATCCACCCAC	Check primer
**Primers used for construction of markerless deletion of *Piso_02323***		
trbNNcoI_F	GCGCccatggGCATGGCCGGTAAGTGGTTG	NcoI
trbNEcoRI_R	GCGCgaattcGCGCTGCTGTTCGAGGTACT	EcoRI
DotLKpn_F	GCGCggtaccTGTACTTGGGGCATATCTG	KpnI
DotLNcoI_R	GCGCccatggCCTCACCATAGCATGCAGA	NcoI
trbN_out_R	CTCCTGAAGTTGTCATTCT	Check primer
DotL_out_F	GCGCCTTCGAGCATAACTA	Check primer
**Primers used for construction of markerless deletion of *dotD***		
2355EcoRI_R	GCGCgaattcTTTCGACATAGGCATAGC	EcoRI
trbN-KpnI_F	GCGCggtaccTTGTACCGCGTAGTCGTC	KpnI
trbN-NcoI_R	GCGCccatggGGTAGCTCACCACGTACT	NcoI
dotC-NcoI_F	GCGCccatggCTGAGCGCCTTTACGACAA	NcoI
trbN_out_F	GGCATTCTGGTTCTGGTTC	Check primer
dotC_out_R	CAATTTCCACACGCGTTC	Check primer
**Primers used for construction of markerless deletion of *Piso_02323, trbN, dotD***		
DotCNcoI_F	GCGCccatggGATGACCCTCGAAGACG	NcoI
DotCEcoRI_R	GCGCgaattcCAGCGGGAAGCTCAGAT	EcoRI
Primers DotLKpn_F and DotLNcoI_R were used to amplify the upstream homology region		
Check primers DotL_out_F and dotC_out_R used to validate the deletion		

Primer name	Sequence	Restriction site/Description
**Primers used for construction of markerless deletion of *Piso_02332, Piso_02333***		
2364-MluI_F	GCGCacgcgtCGCGCTCAAGCAGATTCA	MluI
2364-SphI_R	GCGCgcatgcCGGCCACTTTTTCCCAGTA	SphI
2361-XbaI_F	GCGCtctagaAAAAGGTTAGCGTGCAGC	XbaI
2361-MluI_R	GCGCacgcgtCCTAAAGGTTCTCCAATCCA	MluI
2361_out_F	GAATGTTTGAACGAGGCCC	Check primer
2364_out_R	TCACGCGAATGGAGATGTTG	Check primer
**Primers used for construction of markerless deletion of *Piso_02333***		
2362-MluI_R	GCGCacgcgtTCAGTCCTCGTGCTTTTTG	MluI
2362-XbaI_F	GCGCtctagaCATTTTCATGGTGGCGTGT	XbaI
Primers 2364-MluI_F and 2364-SphI_R were used to amplify the upstream homology region		
Check primers 2361_out_F and 2364_out_R used to validate the deletion		
**Primers used for construction of markerless deletion of the *kib* cluster region**		
2341KpnI_F	GCGCggtaccCAATTATTTTTACCCGCTCG	KpnI
2341NcoI_R	GCGCccatggCGTGGCAGGACTAATCTTT	NcoI
2390NcoI_F	GATTTACTGACCGCCATGGT	NcoI
2390EcoRI_R	GCGCgaattcCCTTGCTTTAACCTGTAGTG	EcoRI
2340_out_F	ACGGCCAAGTTGAGTTACAA	Check primer
2390_out_R	AATTTTACAGCCCAAAACCC	Check primer
**Construction of pGPI-SceI::TetAR**		
TetPstI_R	GCGCCtgcagtCAATCGTCACCCTTTCTCG	PstI
TetPstI_F	GCGCctgcagTCAGCGATCGGCTCGTTGCC	PstI
**Ligation check primers flanking the MCS of pGPI-SceI**		
p44_pGPI_F	AGCTGATCCGGTGGATGAC	
p45_pGPI_R	ACGGTTGTGGACAACAAGC	
p131_pGPI_F	ACTCAAGCGTTAGATGC	

Primer name	Sequence	Restriction site/Description
**Construction of pBBR::32**		
2362-EcoRI_F	GCGCgaattcTTCAATTGCTCCCATCTTC	EcoRI
2362-XbaI_R	GCGCtctagaATCAGTCCTCGTGCTTTTTG	XbaI
**Construction of pBBR::33**		
2363-XbaI_R	GCGCtctagaTTCTGCTTGAGCGCGATC	XbaI
2363-EcoRI_F	GCGCgaattcGGACTGATGCAGATGGGTAA	EcoRI
**Construction of pBBR::32-33**		
Primers 2362-EcoRI_F and 2363-XbaI_R were used to amplify *Piso_02332* and *Piso_02333*		
**Ligation check primers flanking the MCS of pBBR1MCS-2**		
M13For (-21)	TGTAAAACGACGGCCAGT	
M13Rev	CAGGAAACAGCTATGACC	
**Insertion check primers for Tn23**		
tn23_F	GCTGCACCATTCGCGTTA	
tn23_R	GCTGATTCGAGGCGTTAA	
**Construction of pLG99::Km**		
Kan-AatII_F	GCGCgacgtcGTCATTTCGAACCCCAGAG	AatII
Kan-AatII_R	GCGCgacgtcGACTGGGCGGTTTTATGGA	AatII

Table S3. List of Pseudomonads carrying the T4BSS cluster elements and the comparison of the GC content, the nucleotide identity and the query cover shared with IsoF

Strain	GC content (%)			T4BSS gene cluster (%)		Isolated from
	T4BSS cluster	Genome [3]	Difference [4]	Query cover	Identity	
P. putida H8234	60.42	62.32	1.9	60	84.82	Clinical
P. putida BIRD-1	60.41	62.42	2.01	56	84.03	Rhizosphere
P. putida JB	60.36	62.51	2.15	55	84.05	Soil
P. putida N1R	60.36	62.65	2.29	57	84.05	Soil
P. putida S12	60.34	62.51	2.17	59	84.09	Soil
Pseudomonas sp. SWI36	60.36	62.54	2.18	57	85.02	Soil
P. putida KF715	60.28	62.65	2.37	61	84.81	Soil
P. putida B1	60.29	62.69	2.4	59	84.77	Soil
Pseudomonas sp. Irchel s3f19	60.24	62.76	2.52	75	99.67	Soil
P. monteilii TCU-CK1	59.67	62.64	2.97	64	84.8	Soil
P. putida TS312	59.8	62.92	3.12	61	84.8	Paper mill
P. putida IsoF – *kib* cluster [1]	60.09	63.34	3.25	-	-	Rhizosphere
P. putida IsoF – GI [2]	58.76	63.36	4.6	-	-	

[1] The GC percentage of the T4BSS cluster region

[2] The GC percentage of the entire IsoF genomic island (I-GI) including the T4BSS cluster, the Type I RM system and the integrase

[3] Genome GC content % excluding the correspondent T4BSS or T4BSS cluster/I-GI region

[4] Difference between the T4BSS cluster % minus the Genome %

Table S4. List of the 61 genes encoded within the I-GI including *kib* cluster genes and their protein ID accession numbers.

#	Locus_tag	Protein ID	#	Locus_tag	Protein ID
1	PisoF_02313	UPL06638.1	32	PisoF_02344	UPL06669.1
2	PisoF_02314	UPL06639.1	33	PisoF_02345	UPL06670.1
3	PisoF_02315	UPL06640.1	34	PisoF_02346	UPL06671.1
4	PisoF_02316	UPL06641.1	35	PisoF_02347	UPL06672.1
5	PisoF_02317	UPL06642.1	36	PisoF_02348	UPL06673.1
6	PisoF_02318	UPL06643.1	37	PisoF_02349	UPL06674.1
7	PisoF_02319	UPL06644.1	38	PisoF_02350	UPL06675.1
8	PisoF_02320	UPL06645.1	39	PisoF_02351	UPL06676.1
9	PisoF_02321	UPL06646.1	40	PisoF_02352	UPL06677.1
10	PisoF_02322	UPL06647.1	41	PisoF_02353	UPL06678.1
11	PisoF_02323	UPL06648.1	42	PisoF_02354	UPL06679.1
12	PisoF_02324	UPL06649.1	43	PisoF_02355	UPL06680.1
13	PisoF_02325	UPL06650.1	44	PisoF_02356	UPL06681.1
14	PisoF_02326	UPL06651.1	45	PisoF_02357	UPL06682.1
15	PisoF_02327	UPL06652.1	46	PisoF_02358	UPL06683.1
16	PisoF_02328	UPL06653.1	47	PisoF_02359	UPL06684.1
17	PisoF_02329	UPL06654.1	48	PisoF_02360	UPL06685.1
18	PisoF_02330	UPL06655.1	49	PisoF_02361	UPL06686.1
19	PisoF_02331	UPL06656.1	50	PisoF_02362	UPL06687.1
20	PisoF_02332	UPL06657.1	51	PisoF_02363	UPL06688.1
21	PisoF_02333	UPL06658.1	52	PisoF_02364	UPL06689.1
22	PisoF_02334	UPL06659.1	53	PisoF_02365	UPL06690.1
23	PisoF_02335	UPL06660.1	54	PisoF_02366	UPL06691.1
24	PisoF_02336	UPL06661.1	55	PisoF_02367	UPL06692.1
25	PisoF_02337	UPL06662.1	56	PisoF_02368	UPL06693.1
26	PisoF_02338	UPL06663.1	57	PisoF_02369	UPL06694.1
27	PisoF_02339	UPL06664.1	58	PisoF_02370	UPL06695.1
28	PisoF_02340	UPL06665.1	59	PisoF_02371	UPL06696.1
29	PisoF_02341	UPL06666.1	60	PisoF_02372	UPL06697.1
30	PisoF_02342	UPL06667.1	61	PisoF_02373	UPL06698.1
31	PisoF_02343	UPL06668.1			

Table S5. Transposon sequencing results for each treatment

Treatments	Total no. of reads	No. of unique insertion sites	Insertion frequency (bp)	No. of essential genes
Liquid	7,364,199	764,392	7.7	607
Plate	7,054,399	649,830	9.1	616
Mixed	7,419,977	851,838	6.9	644

Table S6. Unique transposon insertions found within the I-GI including *kib* cluster genes. Red highlighted row indicates the gene found to be essential in all treatments.

Locus_tag	Description	Liquid		Solid		Mixed	
		UID[1]	UIC[2]	UID	UIC	UID	UIC
PisoF_02313	hypothetical protein	0.080	21	0.071	19	0.080	21
PisoF_02314	type IV secretion protein DotI/IcmL	0.086	65	0.088	67	0.100	76
PisoF_02315	type IV secretion system protein IcmK/DotH	0.058	59	0.068	69	0.057	58
PisoF_02316	type IV secretion protein DotG/IcmE	0.094	136	0.080	115	0.101	146
PisoF_02317	type IV secretion protein DotF	0.120	75	0.113	70	0.097	60
PisoF_02318	type IV secretion protein DotIE	0.071	42	0.080	47	0.048	28
PisoF_02319	hypothetical protein	0.096	37	0.052	20	0.078	30
PisoF_02320	type IV secretion system protein DotN	0.075	56	0.072	54	0.067	50
PisoF_02321	type IV secretion system protein IcmB/DotO	0.095	292	0.089	273	0.086	263
PisoF_02322	type IV secretion system protein IcmO/DotL	0.064	188	0.060	176	0.058	172
PisoF_02323	hypothetical protein	0.067	42	0.070	43	0.063	39
PisoF_02324	conjugal transfer protein TrbN	0.037	20	0.051	27	0.042	22
PisoF_02325	type IV secretion system protein DotD	0.071	36	0.050	25	0.043	21
PisoF_02326	type IV secretion system protein DotC	0.051	49	0.060	58	0.043	41
PisoF_02327	type IV secretion protein DotB	0.110	132	0.089	107	0.085	102
PisoF_02328	type IV secretion system protein IcmT	0.056	15	0.077	21	0.078	21
PisoF_02329	type IV secretion system protein IcmP/DotM	0.082	109	0.058	76	0.052	69
PisoF_02330	disulfide isomerase/thiol-disulfide oxidase	0.030	29	0.036	35	0.029	28
PisoF_02331	hypothetical protein	0.031	25	0.035	28	0.031	24
PisoF_02332	hypothetical protein	0.009	8	0.002	2	0.006	5
PisoF_02333	hypothetical protein	0.034	20	0.024	14	0.020	11
PisoF_02334	hypothetical protein	0.059	50	0.035	30	0.061	52
PisoF_02335	hypothetical protein	0.034	12	0.048	17	0.047	17
PisoF_02336	hypothetical protein	0.060	36	0.057	34	0.072	43
PisoF_02337	type IV secretion system protein DotA-like protein	0.068	244	0.070	251	0.062	221
PisoF_02338	hypothetical protein	0.101	43	0.110	46	0.113	47
PisoF_02339	hypothetical protein	0.023	10	0.026	12	0.039	18
PisoF_02340	hypothetical protein	0.078	141	0.073	132	0.053	95
PisoF_02341	hypothetical protein	0.062	27	0.053	23	0.042	18
PisoF_02342	hypothetical protein	0.101	127	0.105	132	0.106	133
PisoF_02343	hypothetical protein	0.096	51	0.099	53	0.103	55

Locus_tag	Description	Liquid		Solid		Mixed	
		UID	UIC	UID	UIC	UID	UIC
PisoF_02344	hypothetical protein	0.073	36	0.065	32	0.081	40
PisoF_02345	type IV secretion system protein DotU	0.110	78	0.121	86	0.133	94
PisoF_02346	hypothetical protein	0.064	22	0.086	30	0.059	21
PisoF_02347	hypothetical protein	0.033	20	0.034	21	0.039	24
PisoF_02348	hypothetical protein	0.065	48	0.060	45	0.077	57
PisoF_02349	hypothetical protein	0.104	47	0.116	52	0.088	40
PisoF_02350	hypothetical protein	0.050	21	0.073	31	0.061	26
PisoF_02351	hypothetical protein	0.009	12	0.008	10	0.010	14
PisoF_02352	peptidase M23	0.025	29	0.011	13	0.013	14
PisoF_02353	hypothetical protein	0.040	48	0.027	32	0.035	41
PisoF_02354	hypothetical protein	0.026	30	0.016	18	0.012	14
PisoF_02355	hypothetical protein	0.044	33	0.030	23	0.025	19
PisoF_02356	hypothetical protein	0.072	60	0.065	54	0.070	59
PisoF_02357	type IV secretion protein IcmW	0.074	38	0.069	36	0.054	28
PisoF_02358	hypothetical protein	0.187	401	0.176	379	0.169	364
PisoF_02359	nuclease	0.092	71	0.079	61	0.081	63
PisoF_02360	DNA helicase II	0.165	305	0.145	268	0.151	280
PisoF_02361	putative transcriptional regulator	0.100	20	0.083	17	0.093	19
PisoF_02362	Type I restriction enzyme EcoKI M protein	0.161	250	0.129	201	0.133	207
PisoF_02363	Type I restriction enzyme specificity protein MPN_089	0.100	115	0.079	91	0.069	79
PisoF_02364	anticodon nuclease	0.070	83	0.048	58	0.048	58
PisoF_02365	Type I restriction enzyme EcoR124II R protein	0.126	392	0.119	368	0.117	364
PisoF_02366	putative P-loop ATPase	0.138	248	0.135	242	0.118	212
PisoF_02367	hypothetical protein	0.069	33	0.062	30	0.046	22
PisoF_02368	7-cyano-7-deazaguanine synthase	0.109	143	0.103	135	0.091	119
PisoF_02369	hypothetical protein	0.107	28	0.073	19	0.105	27
PisoF_02370	integrase	0.093	91	0.096	94	0.087	85
PisoF_02371	hypothetical protein	0.182	65	0.200	72	0.172	62
PisoF_02372	hypothetical protein	0.098	137	0.088	123	0.109	153
PisoF_02373	prophage CP4-57 integrase	0.146	184	0.155	195	0.160	201
	Total number of UIC in the I-GI[3]		5283		4912		4793

[1] UID: Unique insertion density

[2] UIC: Unique insertion count

[3] Normalized number of UIC using the number of unique insertion sites from Table 3.

Supplementary references

1. Casadaban, M. J. & Cohen, S. N. Analysis of gene control signals by DNA fusion and cloning in *Escherichia coli*. *J. Mol. Biol.* **138**, 179–207 (1980).
2. Herrero, M., De Lorenzo, V. & Timmis, K. N. Transposon vectors containing non-antibiotic resistance selection markers for cloning and stable chromosomal insertion of foreign genes in Gram-negative bacteria. *J. Bacteriol.* **172**, 6557–6567 (1990).
3. Simon, R., Priefer, U. & Pühler, A. A broad host range mobilization system for in vivo genetic engineering: Transposon mutagenesis in gram negative bacteria. *Bio/Technology* **1**, 784–791 (1983).
4. Hanahan, D. Studies on transformation of *Escherichia coli* with plasmids. *J. Mol. Biol.* **166**, 557–580 (1983).
5. Miller, V. L. & Mekalanos, J. J. A novel suicide vector and its use in construction of insertion mutations: Osmoregulation of outer membrane proteins and virulence determinants in *Vibrio cholerae* requires ToxR. *J. Bacteriol.* **170**, 2575–2583 (1988).
6. Steidle, A. *et al.* Visualization of N-acylhomoserine lactone-mediated cell-cell communication between bacteria colonizing the tomato rhizosphere. *Appl. Environ. Microbiol.* **67**, 5761–5770 (2001).
7. Conway, H. F. *et al. Pseudomonas aureofaciens* Kluyver and phenazine alpha-carboxylic acid, its characteristic pigment. *J. Bacteriol.* **72**, 412–417 (1956).
8. Rakhimova, E., Munder, A., Wiehlmann, L., Bredenbruch, F. & Tümmler, B. Fitness of isogenic colony morphology variants of *Pseudomonas aeruginosa* in murine airway infection. *PLoS One* **3**, e1685 (2008).
9. Rumbaugh, K. P., Griswold, J. A., Iglewski, B. H. & Hamood, A. N. Contribution of quorum sensing to the virulence of *Pseudomonas aeruginosa* in burn wound infections. *Infect. Immun.* **67**, 5854–5862 (1999).
10. Choi, K.-H. & Schweizer, H. P. Mini-Tn7 insertion in bacteria with single attTn7 sites: example *Pseudomonas aeruginosa*. *Nat. Protoc.* **1**, 153–161 (2006).
11. Koch, B., Jensen, L. E. & Nybroe, O. A panel of Tn7-based vectors for insertion of the gfp marker gene or for delivery of cloned DNA into Gram-negative bacteria at a neutral chromosomal site. *J. Microbiol. Methods* **45**, 187–195

(2001).
12. Flannagan, R. S., Linn, T. & Valvano, M. A. A system for the construction of targeted unmarked gene deletions in the genus *Burkholderia*. *Environ. Microbiol.* **10**, 1652–1660 (2008).
13. Figurski, D. H. & Helinski, D. R. Replication of an origin-containing derivative of plasmid RK2 dependent on a plasmid function provided in trans. *Proc. Natl. Acad. Sci. U. S. A.* **76**, 1648–1652 (1979).
14. Kovach, M. E. *et al.* Four new derivatives of the broad-host-range cloning vector pBBR1MCS, carrying different antibiotic-resistance cassettes. *Gene* **166**, 175–176 (1995).
15. Gallagher, L. A. *et al.* Sequence-defined transposon mutant library of *Burkholderia thailandensis*. *MBio* **4**, e00604-13 (2013).
