# Supplementary material for: Pseudomonas putida mediates bacterial killing, biofilm invasion and biocontrol with a type IVB secretion system
Source: Nat Microbiol. 2022 Sep 19;7(10):1547–57. doi: 10.1038/s41564-022-01209-6 (PMC9519443; doi:10.1038/s41564-022-01209-6)
Supplement: Supplementary file 2 — Reporting Summary. [file 41564_2022_1209_MOESM2_ESM.pdf]

nature portfolio

Corresponding author(s): Leo Eberl

Last updated by author(s): Jul 11, 2022

# Reporting Summary

Nature Portfolio wishes to improve the reproducibility of the work that we publish. This form provides structure for consistency and transparency in reporting. For further information on Nature Portfolio policies, see our Editorial Policies and the Editorial Policy Checklist.

## Statistics

For all statistical analyses, confirm that the following items are present in the figure legend, table legend, main text, or Methods section.

| n/a | Confirmed | |
|---|---|---|
| ☐ | ☒ | The exact sample size (*n*) for each experimental group/condition, given as a discrete number and unit of measurement |
| ☐ | ☒ | A statement on whether measurements were taken from distinct samples or whether the same sample was measured repeatedly |
| ☐ | ☒ | The statistical test(s) used AND whether they are one- or two-sided<br>*Only common tests should be described solely by name; describe more complex techniques in the Methods section.* |
| ☒ | ☐ | A description of all covariates tested |
| ☐ | ☒ | A description of any assumptions or corrections, such as tests of normality and adjustment for multiple comparisons |
| ☐ | ☒ | A full description of the statistical parameters including central tendency (e.g. means) or other basic estimates (e.g. regression coefficient) AND variation (e.g. standard deviation) or associated estimates of uncertainty (e.g. confidence intervals) |
| ☐ | ☒ | For null hypothesis testing, the test statistic (e.g. *F*, *t*, *r*) with confidence intervals, effect sizes, degrees of freedom and *P* value noted<br>*Give P values as exact values whenever suitable.* |
| ☒ | ☐ | For Bayesian analysis, information on the choice of priors and Markov chain Monte Carlo settings |
| ☒ | ☐ | For hierarchical and complex designs, identification of the appropriate level for tests and full reporting of outcomes |
| ☒ | ☐ | Estimates of effect sizes (e.g. Cohen's *d*, Pearson's *r*), indicating how they were calculated |

*Our web collection on statistics for biologists contains articles on many of the points above.*

## Software and code

Policy information about availability of computer code

| | |
|---|---|
| Data collection | Leica Application Suite LAS V4.5 and Leica Application Suite LAS AF 2.7.3 (Leica Microsystems, Germany) |
| Data analysis | IMARIS v9.6.0 (Bitplane, Switzerland). PRISM v8.4.1 (GraphPad Software). ImageJ v1.53f51 (Schneider et al., 2012). ClustVis Webtool (Metsalu et al., 2015). MAUVE v2.4.0 (Darling et al., 2004). MultiGeneBlast v1.1..14 (Medema et al., 2013). RaxmlGUI v2.0 (Edler et al 2021) |

For manuscripts utilizing custom algorithms or software that are central to the research but not yet described in published literature, software must be made available to editors and reviewers. We strongly encourage code deposition in a community repository (e.g. GitHub). See the Nature Portfolio guidelines for submitting code & software for further information.

## Data

Policy information about availability of data

All manuscripts must include a data availability statement. This statement should provide the following information, where applicable:

- Accession codes, unique identifiers, or web links for publicly available datasets
- A description of any restrictions on data availability
- For clinical datasets or third party data, please ensure that the statement adheres to our policy

The genome sequence of IsoF has been deposited in NCBI under the accession number CP072013. FASTQ files generated from the Illumina MiSeq platform are publicly available at the NCBI short reads archive (SRA) under the BioProject: PRJNA730700. Individual datasets have following accession numbers: Liquid: SRR14612110, Plate: SRR14612109 and Mixed: SRR14612108.

# Field-specific reporting

Please select the one below that is the best fit for your research. If you are not sure, read the appropriate sections before making your selection.

☒ Life sciences ☐ Behavioural & social sciences ☐ Ecological, evolutionary & environmental sciences

For a reference copy of the document with all sections, see nature.com/documents/nr-reporting-summary-flat.pdf

# Life sciences study design

All studies must disclose on these points even when the disclosure is negative.

| | |
|---|---|
| Sample size | We did not perform a sample-size calculation but made sure that a sufficient number of replicates were taken following previous experience with these types of experimental data. Furthermore, the detailed statistical analysis of the data generated in this study indicated a high level of reproducibility. Specifically, all competition experiments were performed at least as biological triplicates (exact number of replicates indicated by dots) and each replicate measure comprised of 2 technical replicates. In the monolayer competition experiments, the number of dead cells was determined by at least 3 technical replicates. For the flow cell biofilm experiments, up to 3 biological replicates were carried out and each replicate measurement comprised 3 technical replicates. In planta experiments were performed in biological triplicate, using a minimum of 8 plants per treatment for each replicate. |
| Data exclusions | No data were excluded from the analysis. |
| Replication | Experiments were performed at least in triplicate. The Tn-seq analysis was carried out only once for each of the three growth regimes, as we used a highly saturated transposon library containing more than 700.000 insertion mutants, i.e. an insertion every 8 bp. By using a large mutant number as inoculum we made sure that no bottlenecks were created in our experiments, which otherwise may have create a bias. It is also noteworthy that all three growth regimes identified that same genes and thus can be considered biological replicates. |
| Randomization | Randomization is not relevant for our study that investigated interactions between bacteria and bacteria and their host plant. |
| Blinding | Blinding is not relevant for our study that investigated interactions between bacteria and bacteria with their host plant. |

# Reporting for specific materials, systems and methods

We require information from authors about some types of materials, experimental systems and methods used in many studies. Here, indicate whether each material, system or method listed is relevant to your study. If you are not sure if a list item applies to your research, read the appropriate section before selecting a response.

## Materials & experimental systems

| n/a | Involved in the study | |
|---|---|---|
| ☒ | ☐ | Antibodies |
| ☒ | ☐ | Eukaryotic cell lines |
| ☒ | ☐ | Palaeontology and archaeology |
| ☒ | ☐ | Animals and other organisms |
| ☒ | ☐ | Human research participants |
| ☒ | ☐ | Clinical data |
| ☒ | ☐ | Dual use research of concern |

## Methods

| n/a | Involved in the study | |
|---|---|---|
| ☒ | ☐ | ChIP-seq |
| ☒ | ☐ | Flow cytometry |
| ☒ | ☐ | MRI-based neuroimaging |
