# Supplementary figures and images for: Pseudomonas putida mediates bacterial killing, biofilm invasion and biocontrol with a type IVB secretion system
Source: Nat Microbiol. 2022 Sep 19;7(10):1547–57. doi: 10.1038/s41564-022-01209-6 (PMC9519443; doi:10.1038/s41564-022-01209-6)

Source Data Extended Data Fig. 6

SDS-PAGE Protein gel, Commasie Brilliant Blue staining

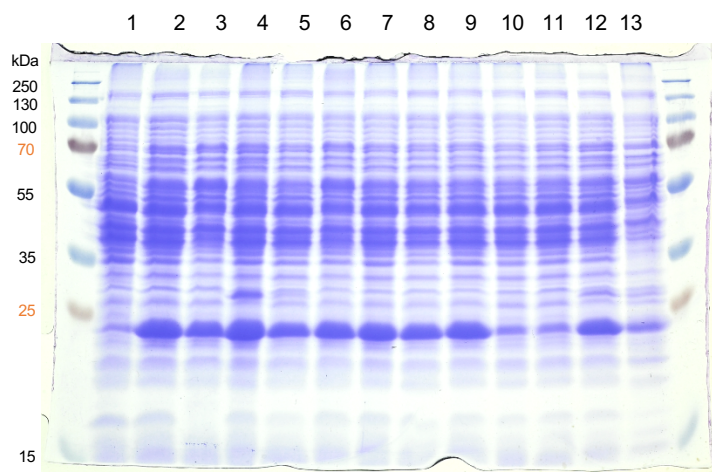

Supplement: Source Data Extended Data Fig. 6 — Unprocessed SDS gel. [file 41564_2022_1209_MOESM16_ESM.pdf]
